# What helminth genomes have taught us about parasite evolution

**DOI:** 10.1017/S0031182014001449

**Published:** 2014-12-08

**Authors:** MAGDALENA ZAROWIECKI, MATT BERRIMAN

**Affiliations:** Parasite Genomics, Wellcome Trust Sanger Institute, Wellcome Trust Genome Campus, Hinxton, Cambridge CB10 1SA, UK

**Keywords:** parasite genomics, phylogeny, comparative transcriptomics, evolution of parasitism, Cestoda, Trematoda, Nematoda

## Abstract

The genomes of more than 20 helminths have now been sequenced. Here we perform a meta-analysis of all sequenced genomes of nematodes and Platyhelminthes, and attempt to address the question of what are the defining characteristics of helminth genomes. We find that parasitic worms lack systems for surface antigenic variation, instead maintaining infections using their surfaces as the first line of defence against the host immune system, with several expanded gene families of genes associated with the surface and tegument. Parasite excretory/secretory products evolve rapidly, and proteases even more so, with each parasite exhibiting unique modifications of its protease repertoire. Endoparasitic flatworms show striking losses of metabolic capabilities, not matched by nematodes. All helminths do however exhibit an overall reduction in auxiliary metabolism (biogenesis of co-factors and vitamins). Overall, the prevailing pattern is that there are few commonalities between the genomes of independently evolved parasitic worms, with each parasite having undergone specific adaptations for their particular niche.

## INTRODUCTION

Parasitic worms (helminths) cause some of the most devastating threats to human health and livelihoods. Soil-transmitted helminths (STHs) cause neglected tropical diseases affecting >1 billion people worldwide (Bethony *et al.* 2006), blood flukes (schistosomes) infect more than 200 million people (Steinmann *et al.* 2006) and the global tapeworm disease burden has been estimated at 1 million disability-adjusted life years (Budke *et al.* 2009). Apart from causing human mortality and disability, parasitic worm infections also threaten food security; larval tapeworm infections (echinococcosis) of livestock cause annual losses of US$2 billion in US cattle alone (Torgerson and Macpherson, 2011), and US$80 billion of annual crop damage is caused by plant parasitic nematodes (Nicol *et al.* 2011). Next generation DNA sequencing is now providing an unparalleled opportunity to deepen our understanding of how parasites’ genomes have been affected by adaptation to parasitism. This review will conduct a meta-analysis of the more than 30 genome sequences from nematodes and flatworms currently available. We will discuss the progress of genomics in parasitic worms, and review any common themes in genome structure and content of parasitic worms.

**Fig. 1.**
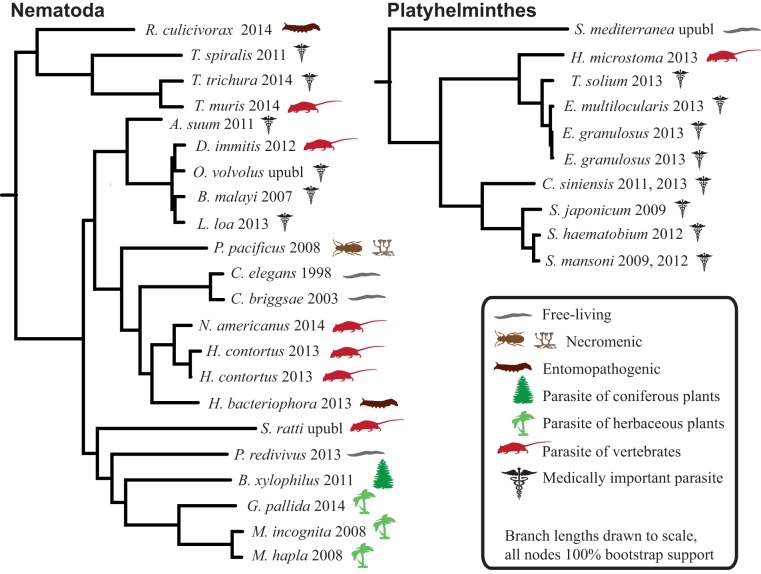
Helminth phylogenies show that animal and plant parasitism has evolved on several occasions in nematodes, exhibiting a wide variety of hosts and parasitic strategies. The species name is followed by the year that genome was published, and the species mode of parasitism.

Humans are parasitized by two major groups of parasitic worms; the Nematoda (roundworms) and Platyhelminthes (flatworms). Within flatworms endoparasitism is believed to have arisen only once (Littlewood *et al.* 1999; Hahn *et al.* 2014), with all species being parasites of animals, typically with one invertebrate and one vertebrate host (Fig. 1, Supplementary Table 1). In nematodes, both plant and animal parasites have evolved in several lineages, infecting a large spectrum of hosts (Blaxter *et al.* 1998; Dieterich and Sommer, 2009; Blaxter and Koutsovoulos, 2014). Searching for common themes is challenging; both because of the few instances parasitism has evolved in worms, and also because the diversity of hosts and niches that parasites occupy (Fig. 1). At the morphological level it is apparent that there are some common themes between even very disparate parasitic groups. For instance, the simplification of external morphology (reduction of pigmentation, simplified body shape), and reduction of sensory inputs (visual and chemosensory organs, and the capability of neuronal processing of those inputs). It could thus be reasonable to hypothesize that parasitic worms would exhibit genomic regression mirroring their morphological regression. All parasites would also by necessity have to evolve methods for host invasion and host immune system evasion, and many exhibit increased reproductive output.

On the other hand, many adaptations to parasitism are more specific, such as the metacestode in some tapeworms (a whole new life stage) and the stichosome in whipworms (a long slender organ for intracellular feeding from host-cells). Adaptation might in some cases be divergent instead of convergent, for instance if each parasite adapt their metabolism to fit the nutrients available in the host, but the metabolite availability varies greatly between hosts (e.g. between invertebrates and vertebrates, or woody and herbaceous plants). Even when adaptation occurs to common environments the genomic underpinnings could be different.

We thus have two alternative hypotheses: (1) that because of the diversity of evolutionary starting points (proto-parasites), and the hosts to which they adapt, each independently evolved parasitic clade has unique adaptations in its genomes and gene content; (2) that there are common genomic adaptations in independently evolved parasitic worms. These hypotheses are not mutually exclusive, as both could be true for various systems. However, whereas the latter scenario (common adaptations) has been repeatedly shown for morphological features and life-history traits (Quicke and Belshaw, 1999; Poulin, 2011), there are few (if any) examples of genomic convergence amongst helminths, although it is known to have occurred in many organisms (Christin *et al.* 2010). We will here review the evidence of convergent and unique adaptations respectively, in currently available helminths genomes.

### Nematode genomes are diverse

The first animal genome ever published was from the free-living nematode *Caenorhabditis elegans*, and it was accompanied by an ambitious programme for functional characterization of genes (*C. elegans* Sequencing Consortium, *1998*). That genome remains the point of reference for all subsequent genome sequencing efforts of nematodes, free-living and parasites alike. It has been joined by the genomes of the other free-living nematodes *Caenorhabditis briggsae* (Stein *et al.* 2003) and *Panagrellus redivivus* (Srinivasan *et al.* 2013).

When the first genome of a parasitic nematode was sequenced – the filarial nematode *Brugia malayi –* the ~350 million years of separate evolution from *C. *elegans** and *C. briggsae* meant that there were 3979 gene clusters shared between *C. elegans, C. briggsae, B. malayi* and a fruit fly (representing animal core proteins), but only 174 clusters which had members of all nematode species (ubiquitous and nematode specific) (Ghedin *et al.* 2007). Further genome sequencing of nematodes has reinforced this understanding that nematode genomic diversity is vast, and that multiple reference nematode genomes are needed (Kumar *et al.* 2012).

Soon after that sequences of plant-parasitic nematode genomes started to emerge: root-knot nematodes *Meloidogyne incognita* (Abad *et al.* 2008) and *Meloidogyne hapla* (Opperman *et al.* 2008), the pine-wilt nematode *Bursaphelenchus xylophilus* (Kikuchi *et al.* 2011) and more recently the potato cyst nematode *Globodera pallida* (Cotton *et al.* 2014). Sequencing the very minimalist genome of *M. hapla*, with 5000 fewer genes than *C. elegans* (Opperman *et al.* 2008), raised the possibility that parasite genomes might be smaller and have fewer genes than those of free-living species. However, as the number of sequenced genomes has increased, the theory has been short-lived, with genome size and organization of parasitic helminths being just as diverse as those of free-living species (Table 1), as also observed previously (Bird *et al.* 2014).

**Table 1.** Summary of sequenced worm genomes

Species	Clade	Genome size (Mb)	Scaffold N50 (Mb)	Scaffold N50 (#)	Scaffolds	Number of protein-coding genes	Author	Source	Type of parasitism
Nematodes								
*Ascaris suum*	Ascaridida III	273	0·407	–	1618	18 542	Jex *et al.* (2011)	WormBase	V
*Brugia malayi*	Spirurida III	96	0·094	–	8180	11 515	Ghedin *et al.* (2007)	WormBase	I/V
*Bursaphelenchus xylophilus*	Tylenchida IV	75	1·16	–	1231	18 074	Kikuchi *et al.* (2011)	GeneDB	P
*Caenorhabditis briggsae*	Rhabditia V	108	0·475	–	899	19 507	Stein *et al.* (2003)	WormBase	NA
*Caenorhabditis elegans*	Rhabditia V	100	14·3	3	7	19 099	*C. elegans* Sequencing Consortium, (1998)	WormBase	NA
*Dirofilaria immitis*	Spirurida III	84	0·011	–	31 291	10·179	Godel *et al.* (2012)	WormBase	I/V
*Globodera pallida*	Tylenchida IV	125	0·122	–	6873	16 419	Cotton *et al.* (2014)	WormBase	P/F
*Haemonchus contortus*	Rhabditia V	320	0·056	–	–	23 610	Schwarz *et al.* (2013)	WormBase	V/F
*Haemonchus contortus*	Rhabditia V	370	0·083	–	26 044	21 799	Laing *et al.* (2013)	Sanger FTP	V/F
*Heterorhabditis bacteriophora*	Rhabditia V	77	0·312	–	1263	21 250	Bai *et al.* (2013)	WormBase	I/F
*Loa loa*	Spirurida III	91	0·172	–	5774	14 907	Desjardins *et al.* (2013)	WormBase	I/V
*Meloidogyne hapla*	Tylenchida IV	54	0·083	–	1532	14 420	Opperman *et al.* (2008)	WormBase	P/F
*Meloidogyne incognita*	Tylenchida IV	86	0·593	–	–	19 212	Abad *et al.* (2008)	www6.inra.fr/meloidogyne_incognita	P/F
*Necator americanus*	Rhabditia V	244	0·213	283	11 713	19 151	Tang *et al.* (2014)	GenBank	Bacterivore/V
*Onchocerca volvulus*	Spirurida III	NA	–	–	–	–	Unpublished	WormBase	I/V
*Panagrellus redivivus*	Rhabditia V	64	0·262	–	–	24 249	Srinivasan *et al.* (2013)	WormBase	NA
*Pristionchus pacificus*	Rhabditia V	169	0·169	–	2894	29 201	Dieterich *et al.* (2008)	WormBase	I/F
*Romanomermis culicivorax*	Dorylaimia II	323	0·0176		62 537	48 171	Schiffer *et al.* (2013)	nematodes.org/genomes/romanomermis_culicivorax/	I/F
*Strongyloides ratti*	Rhabditia V	–	–	–	–	–	Unpublished	WormBase	V/F
*Trichinella spiralis*	Dorylaimia II	64	1·7	9	8795	15 808	Mitreva *et al.* (2011)	WormBase	V/V
*Trichuris muris*	Dorylaimia II	85	1·58	15	1123	11 004	Foth *et al.* (2014)	GeneDB	V
*Trichuris trichiura*	Dorylaimia II	73	0·071	263	3711	9650	Foth *et al.* (2014)	Sanger FTP	V
Flatworms								
*Clonorchis sinensis*	Trematoda	547	0·233	–	6190	13 634	Huang *et al.* (2013)	fluke.sysu.edu.cn	I/V
*Clonorchis sinensis*	Trematoda	516	0·043	–	26 466	16 258	Wang *et al.* (2011)	NA	I/V
*Echinococcus granulosus*	Cestoda	152	0·68	–	967	11 325	Zheng *et al.* (2013)	GenBank	V/V
*Echinococcus granulosus*	Cestoda	115	5·2	6	–	10 231	Tsai *et al.* (2013)	GeneDB	V/V
*Echinococcus multilocularis*	Cestoda	115	13·8	4	–	10 345	Tsai *et al.* (2013)	GeneDB	V/V
*Hymenolepis microstoma*	Cestoda	141	0·5	75	–	10 241	Tsai *et al.* (2013)	GeneDB	V/I
*Schistosoma haematobium*	Trematoda	385	0·307	365	–	13 073	Young *et al.* 2012	schistodb.net	I/V
*Schistosoma japonicum*	Trematoda	397	0·177	–	–	13 469	Zhou *et al.* (2009)	GeneDB	I/V
*Schistosoma mansoni*	Trematoda	363	0·832	–	–	11 809	Berriman *et al.* (2009)	NA	I/V
*Schistosoma mansoni*	Trematoda	364	2	–	885	10 852	Protasio *et al.* (2012)	GeneDB	I/V
*Schmidtea mediterranea*	Turbellaria	NA	–	–	–	–	Unpublished	NA	NA
*Taenia solium*	Cestoda	122	0·07	439	–	12 490	Tsai *et al.* (2013)	GeneDB	V/V

The statistics are extracted from the genome papers, and may not correspond with the data utilized, or statistics reported by other sources.

Systematic classification according to (Blaxter *et al.* 1998) reported.

Type of parasitism: I, invertebrate host; V, vertebrate host; P, plant parasitic; F, free-living.

The necromenic species *Pristionchus pacificus* remains inactive inside the host until the host dies, so its genome may offer key pieces of the puzzle in understanding the evolution of parasitism (Dieterich *et al.* 2008). The *P. pacificus* genome has been followed by those of the more active entomopathogenic parasites *Heterorhabditis* bacteriophora (killing its insect host by regurgitating toxic bacteria into its body cavity) (Bai *et al.* 2013) and *Romanomermis culicivorax* (eating its mosquito host from the inside, before escaping by rupturing the host cuticle) (Schiffer *et al.* 2013), giving us an insight into very diverse parasitic niches. The characterized genomes of animal parasites now also includes more than a dozen species of medical and veterinary importance, including the large roundworm (*Ascaris suum*), the barber's pole worm (*Haemonchus contortus*), the dog heartworm (*Dirofilaria immitis*) and the human hookworm (*Necator americanus*) (Table 1). Available pre-publication are the genomes of *Strongyloides ratti*, *Onchocerca volvulus*, and the filarial nematodes *Acanthocheilonema viteae, Litomosoides sigmodontis* and *Onchocerca ochengi* (on WormBase and www.nematodes.org).

### Flatworm genomes are reduced

Although ectoparasitism has probably evolved several times within flatworms (phylum Platyhelminthes), there is broad consensus that all endoparasitic flatworms are monophyletic (Littlewood *et al.* 1999; Hahn *et al.* 2014). Most closely related to some ectoparasitic parasites (monogeneans), endoparasitic Platyhelminthes form two separate clades; Trematoda (flukes) and Cestoda (tapeworms) (Fig. 1) (Littlewood *et al.* 1999). The members of these endoparasitic clades exhibit some striking examples of morphological regression, with the most extreme examples of some tapeworms having lost a gut, light-sensory organs, pigmentation and all free-living life stages, all of which are thought to have been present in their ancestors.

Flatworms belong to the super-phylum Lophotrochozoa, which also includes molluscs, earthworms and other less well-known phyla. For this group of animals there is a lack of highly finished and well-characterized reference genome, equivalent to *C. elegans* in nematodes. This provides an obstacle to understanding which genes are parasite-specific, pan-lophotrochozoan or pan-Platyhelminth. In fact, the first lophotrochozoan genomes ever characterized were those of parasites; the human blood flukes *Schistosoma mansoni* and *Schistosoma japonicum* (Berriman *et al.* 2009; Zhou *et al.* 2009). These first genomes of schistosomes were followed by more trematodes; *Schistosoma haematobium* (Young *et al.* 2012), the human liver fluke *Clonorchis sinensis* (Wang *et al*. 2011; Huang *et al.* 2013), and a re-assembly of *S. mansoni* (Protasio *et al.* 2012). More recently, the first genomes of tapeworms (Tsai *et al.* 2013; Zheng *et al.* 2013) and a monogenean (*Gyrodactylus salaris*) (Hahn *et al.* 2014) were published. Both the *S. mansoni* and *Echinococcus multilocularis* genomes have been extensively improved and are mostly assembled into chromosomes. That renders their genomes some of the most correct and complete animal genomes ever published (Table 1). Compared to *C. elegans*, there is however still a lack of exhaustive functional characterization of genes. Some information is provided by the draft genome of *Schmidtea mediterranea* with associated RNAi phenotypes and expression patterns (Robb *et al.* 2008). This free-living flatworm is however in some instances too evolutionarily removed from parasitic flatworms to provide useful functional information.

## ESTABLISHING AN INFECTION

The initial invasion of a host is a critical step for parasites, and is underpinned by numerous adaptations. Many parasites have secretions for penetrating host tissues, others rely on host-specific signals for their development. For instance, host bile is a trigger for tapeworms to emerge from the protoscolex (Zheng *et al.* 2013). Through genome and transcriptome sequencing, insights into the molecular basis of host colonization are being uncovered.

### Secreted proteins can be effectors

A natural place to search for parasite genes involved in host invasion are amongst the parasite excretory/secretory (ES) proteins (Hewitson *et al.* 2009). ES proteins include many types of genes; those with antigenic properties, those allowing the parasite to penetrate, digest or modify host tissue and genes allowing the parasite to defend itself against the host immune system. ES proteins are generally identified by a signal peptide that directs the nascent peptide after translation into the secretory pathway. Although signal peptides can be identified bioinformatically based on their hydrophobicity and putative cleavage motifs, such analyses have limitations: (1) gene models may not be complete and correct at the start, so the predicted gene start may not contain the true signal peptide; (2) proteins without a traditional signal peptide can be secreted through alternative (sometimes unidentified) secretion pathways; (3) signal-peptides do not reveal where in an organism proteins are excreted/secreted, so secreted proteins may remain in intracellular vesicles, or inside parasite body cavities, and never be exposed to the host.

Amongst proteins excreted/secreted externally of the parasite, there are some which do not have the specific purpose of interacting with the host, and some which are true ‘effectors’ – protein secreted by the parasite in order to manipulate the host. Lists of genes that contain ES proteins are commonly published in genome papers, but because of the above restrictions, they only reveal potential effector proteins. For the *A. suum* and *S. haematobium* genomes a slightly more ambitious approach was taken, identifying orthologues of known immunomodulatory proteins (Jex *et al.* 2011; Young *et al.* 2012).

The presence/absence of a signal peptide does not necessarily affect the function of the protein, so modifications of signal peptidases are fairly frequent, and large gene families often contain both secreted and non-secreted members, i.e. a family of S01A proteases in *Trichuris muris* (Foth *et al.* 2014). Some genes commonly have signal peptides (proteases, proteins involved in neuronal signalling and thioredoxins), while a large proportion of ES proteins differ between species (Supplementary Table S2·1). In order to search for commonalities in function between ES products in parasites, we looked for over-represented annotated functions in proteins with signal peptides, using the Gene Ontology (GO) (Ashburner *et al.* 2000), aware that results may be biased by that not all parasite-specific secreted proteins are annotated with GO-terms. We found that there is very little correlation between GO-terms enriched in ES products and the phylogeny of the species, and hardly any GO-terms are significantly enriched in parasites (Supplementary Table S2·2). This suggests that rapid gene family expansions and switches in secretory capacity occur frequently, as adaptations to specific niches. This has previously been noted in plant-parasitic nematodes, where each taxa displays unique modifications of their effectors (Kikuchi *et al.* 2011; Cotton *et al.* 2014) (for effectors characterized in *Meloidogyne, B. xylophilus* and *G. pallida* orthologous genes had been lost, or had lost the signal peptide).

The possible function of secreted proteins can be further characterized by investigating life stage-specific expression; many ES proteases have significantly different expressions between the free-living and parasitic life stages (Schwarz *et al.* 2013). The invasion process itself has been investigated in *S. mansoni*, where 1518 transcripts were differentially expressed between the infective cercariae, and the schistosomula 3 h after infection (Protasio *et al.* 2012). One hundred and twenty-seven of these proteins are predicted to be secreted, including 18 proteases/protease inhibitors (Supplementary Table S3·1). Likewise, the invasion process in the plant parasitic nematode *G. pallida* showed 612 upregulated and 831 downregulated genes in the transition of the infective J2 life-stage to the parasitic J3 life-stage, including 117 proteins which were secreted, upregulated during invasion life-stages, and may represent novel effector candidates (Cotton *et al.* 2014). These are good examples of how genome and RNA-Seq sequencing can produce short-list of genes potentially important for host invasion.

### Proteases can aid host invasion

Proteases/peptidases are often indicated to have important roles in parasitism (Hewitson *et al.* 2009), displaying functions in metabolism, signalling and protein degradation. From the amino acid sequence it is very difficult to predict whether a protease orthologue is functional, which substrate it works on, and its function(s) in a living organism. Even very similar proteases can act on distinctly different substrates, and in different cellular contexts. Thus, the proteases encoded by a genome are often reported, but the exact consequences of apparent expansions or losses can rarely be discussed in any great detail, e.g. (Zhou *et al.* 2009; Wang *et al*. 2011; Tsai *et al.* 2013). Investigating the timing of expression can add important clues to the function of proteases. *Haemonchus contortus* exhibits a remarkable diversity of secreted proteases, some of which are upregulated in the animal parasitic L4 life stage (Laing *et al.* 2013; Schwarz *et al.* 2013). In *N. americanus*, more than 120 protease genes are upregulated in the blood-feeding stage, including many secreted proteases. In *A. suum*, transcripts encoding secreted peptidases of families M12 (astacins), S9, S33, C1 and C2 are abundantly represented (Jex *et al.* 2011).

We find that no protease family is significantly associated with parasitism (in all species of parasites) (Supplementary Table S4). However, we note a candidate for a repeatedly utilized protease; the M8 metallopeptidase major surface protease (MSP or GP63). It was first identified in *Leishmania* promastigotes, where it has been shown to facilitate migration through the host extracellular layer, as well as affecting AK, MAP and IRAK-1 kinase signalling pathways (McGwire *et al.* 2003; Isnard *et al.* 2012). Expansions of MSPs in helminths was first recognized in *S. japonicum* (Zhou *et al.* 2009), but exist in all schistosomes, as well as in the nematodes *H. contortus* and *N. americanus* (Supplementary Table S5·1, S6; Pfam family Peptidase_M8). Further studies of the functions of helminth MSPs are needed to clarify MSPs functions in helminths.

Overall, there is remarkably little conservation in which protease families are expanded, even within smaller clades (Supplementary Table S4). Even when several parasites exhibit expansions of the same protease family, they are independent occurrences. The relatively high abundance of eukaryotic aspartyl proteases (Pfam Asp, MEROPS A01) in parasitic compared to free-living nematodes is due to several independent expansions, and AO1 is not significantly enriched in parasites overall (Supplementary Table S4). The A01 expansions in *B. xylophilus* and *C. sinensis* are also associated with elevated diversity. The family is particularly expanded in *H. contortus*, where members exist in co-linear clusters, indicating that they arose through recent gene duplication (Laing *et al.* 2013).

**Fig. 2.**
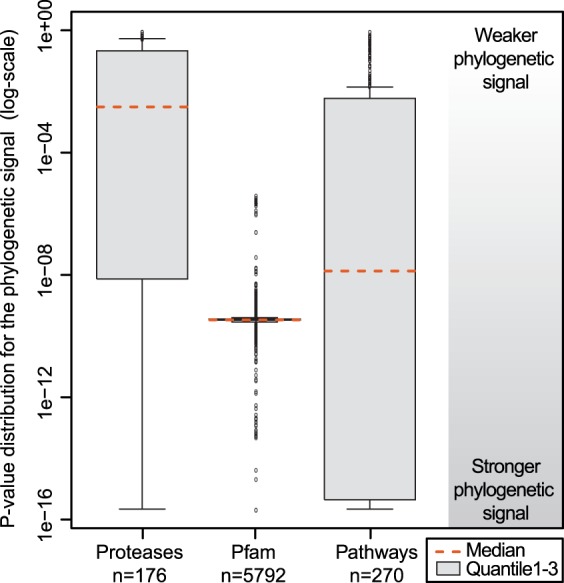
The *P*-value distributions for the phylogenetic signal in the protease, Pfam domain and pathway datasets reveal that the phylogenetic signal is significantly different between all datasets.

While many gene families show a gradual expansion across larger clades, the pattern in proteases is one of local rapid expansions in a small set of species. Genome sequencing has for instance revealed a major expansion in Trichocephalida of S01A trypsin-like proteases (Foth *et al.* 2014), an expansion of M13 and A01 families in *B. xylophilus* (Kikuchi *et al.* 2011), large expansions of C19 in *G. pallida*, and T03 in *C. sinensis* (Supplementary Table S4). This pattern holds true across all the observed species, demonstrated by a remarkable lack of correlation between protease abundance and phylogeny exhibited by many families (Fig. 2, Supplementary Table S4). This is in contrast to the other datasets; using a paired *t*-test (proteases *vs* Pfam *P*-value 3·482e-13, proteases *vs* pathways 2·172e-05, Pfam *vs* pathways 9·412e-09) we found that the phylogenetic signal is significantly weaker in proteases than in domains and pathway representation. We hypothesize that this may indicate that rather than evolving gradually, evolution in protease gene families is driven by accidental fortuitous encounters with off-target substrates (for instance by host switching, or by changing its localization signal). The acquisition of a new function is then followed by multiple gene duplications and perhaps secondary functional divergence, as indicated by the different expression patterns exhibited by copies of A01 in *H. contortus*, and S01 in *T. muris* (Laing *et al.* 2013; Foth *et al.* 2014). If the target then disappears (through another environmental switch, or through host adaptation), proteases without a target are rapidly lost again. Closer functional characterization of expanded proteases is needed to ascertain the drivers of this unusual evolutionary pattern.

## MAINTAINING A CHRONIC INFECTION

Many parasitic worms can live within their host for decades, without getting expelled, and without causing excessive pathology. Recorded cases include patients with more than 30 years of *S. mansoni* infection (Harris *et al.* 1984), and a record 53 years for *E. granulosus* infection (Spruance, 1974). While many single-cell parasites can maintain infections by antigenic variation, there is little evidence for that in helminths (see below). There is however no doubt that helminths can efficiently manipulate the host immune system; inducing an overall suppression of the immune system, inducing strong regulatory T (T_Reg_)-cell activity, and a relative increase of TH2 immune response to TH1 response (Maizels and Yazdanbakhsh, 2003). This allows the parasite to both minimize the inflammation caused, and avoid pathology from developing, resulting in an uneasy truce where the hosts efforts to expel the parasite decreases and infection becomes chronic.

### Absence of classical antigenic variation

For some bacteria, and single-cell parasites such as *Trypanosoma* and *Plasmodium*, the genome structure plays a central role in parasitism (Barry *et al.* 2005; Lemieux *et al.* 2013). Most strikingly, the subtelomeric regions contains large and diverse gene families involved in antigenic variation, allowing the parasite to change which proteins it reveals to the immune system. Particularly in the case of *Trypanosoma brucei* and *Plasmodium falciparum*, the subtelomeric location allows for controlled and mutually exclusive expression of antigens, critical for ensuring that the antigenic repertoire is not exhausted by exposing it to the immune system too soon. Recombination may occur at a higher rate in subtelomeres allowing the parasite to generate new antigenic diversity. For parasitic worms it has been difficult to characterize subtelomeric and other repetitive regions, as draft genomes often fail to appropriately represent them. One of the most contiguous genome assemblies of a parasitic worm, that of *E. multilocularis* (Tsai *et al.* 2013), shows that no genes are over-represented in subtelomeric regions, except for the heat-shock protein 70-like (hsp70-like) gene family, with at least 40% of *E. multilocularis* hsp70-like genes being subtelomeric. The function of these atypical hsp70-like genes remains to be determined (Koziol *et al.* 2009) but each copy lacks the characteristic C-terminal motif of canonical hsp70 copies suggesting an altered function. Different life stage expression patterns and elevated sequence diversity also hints to functional significance (Tsai *et al.* 2013; Zheng *et al.* 2013). Most copies do not have signal peptides or transmembrane domains (Supplementary Table S7), indicating that the majority are probably not displayed on the surface.

Another set of genes with conspicuous gene structure – the >45 micro exon genes (MEGs) found in schistosomes (Berriman *et al.* 2009), which also seem to be present in tapeworms (Tsai *et al.* 2013). These genes appear to be designed to encode high protein diversity; numerous short internal exons, each with a number of bases that divisible by three, enable a huge set of alternative splice forms to be easily generated by exon skipping. Most of the MEGs carry a signal peptide, indicating that they are secreted (Berriman *et al.* 2009), (Supplementary Table S8) and have a high level of sequence divergence between copies, such that some lack any conserved elements at all. No MEG contains any known domains, and their function(s) has not been elucidated.

Another diverse and secreted family are the SCP/TAPS family of protease inhibitors. They seem to have two independent radiations in animal-parasitic nematodes, and are particularly abundant in *N. americanus*, where many are also upregulated in the adult life stage (Tang *et al.* 2014). Although not expanded in parasitic flatworms, the SCP/TAPS (VALs) are also there thought to be important for host–parasite interactions (Chalmers *et al.* 2008). The exact functions of SCP/TAPS in parasitic worms remain to be elucidated, and they are likely to have a diverse set of functions, just as they do in other animals (Cantacessi *et al.* 2009).

Classical antigenic variation genes belong to large gene families, are highly and serially expressed, and localize to the cell surface (Reid, 2014). In spite of extensive searches, and some candidates (MEGS, hsp70 s and SCP/TAPS), as well as galectin-4 and galectin-9 from *A. suum* (Jex *et al.* 2011), it appears that antigenic variation in the same sense as for unicellular parasites does not exist in parasitic worms. Some of the above-mentioned genes may still have primary roles as immunomodulators, or have antigenic or immunomodulatory properties as a side-effect of being highly expressed and secreted at infective life stages.

### Protection against the host through surface modifications

Despite the lack of antigenic variation in parasitic worms, their surfaces remain a vital site for defence against the host immune system, where helminths can utilize alternative (non-protein based) physical and molecular ways of controlling its antigen exposure, or increase their membrane turnover (Fonseca *et al.* 2012). One main strategy to avoid the host immune system appears to be parasite encystment and encapsulation, with the parasite modifying its cuticle, secreting an external layer, or inducing the host to encapsulate it. The molecular mechanisms of such methods are as yet quite poorly understood, but the genome sequences have produced several candidates worth investigating further. Firstly, in the nematode *H. contortus*, the cuticle is significantly re-modelled during the transition from its free-living to parasitic life stage, and 28 collagen genes also exhibited significant differential expression during that transition, along with a set of other cuticular proteins (Laing *et al.* 2013; Schwarz *et al.* 2013). Secondly, the genomes of the tapeworms *Echinococcus granulosus* and *E. multilocularis* are very similar (Tsai *et al.* 2013), but the cysts they produce are not. It is therefore striking that some of the (very few) genes that differ between these species are members of the apomucin family (which are part of the laminated layer) and galactosyltransferases (which probably decorates the apomucins with galactose) (Tsai *et al.* 2013). These galactose modifications have been hypothesized to prevent antibody recognition (Diaz *et al.* 2011). Thirdly, the surface of all adult endoparasitic flatworms is a highly specialized tegument composed of a syncytium attached to an acellular layer, hence their Latin name; Neodermata (meaning ‘new skin’). Some expanded gene families that could be instrumental in providing the toughness and versatility of that skin include the cadherins (which tether adjoining cells together) and tetraspanins (involved in tegument stability) (Tran *et al.* 2010; Tsai *et al.* 2013; Zheng *et al.* 2013). Interestingly, the cadherin family is also more frequent in the animal-parasitic nematodes of all clades, than in their free-living relatives (Supplementary material S5·2), perhaps pointing to a more general parasite adaptation. These preliminary data seem to indicate that in general the genes used are as diverse as the methods of encapsulation, but also that some generally used surface proteins (cadherin, tetraspanins, collagen and apomucin) often get co-opted into creating new parasite-specific morphological structures.

### Redox systems of parasites

It has been suggested that hosts use oxidative stress as means of combating parasites (Schirmer *et al.* 1987), and it was hypothesized that parasites thus would have a very well-developed redox system to defend itself against reactive oxygen species (ROS) attacks. In necromenic *P. pacificus* there were relatively high numbers of detoxification and degradation enzymes compared to free-living *C. elegans* (Dieterich *et al.* 2008) (for instance an increase in P450 copies cytochrome P450 enzymes, glycosyltransferases, sulphotransferases and ATP-binding cassette (ABC) transporters), and these were hypothesized to represent a pre-adaptation for parasitism (Dieterich and Sommer, 2009). After further genome sequencing, it now appears that most of the endoparasites instead have a much reduced set of redox proteins; in both *M. incognita* and tapeworms there is a reduction of P450 s (Abad *et al.* 2008; Tsai *et al.* 2013), and the pattern that animal parasites have less P450 s than their free-living and plant-parasitic relatives remains true when the analysis is expanded to all genomes (Supplementary Table S5·2). *Meloidogyne incognita* has lost glutathione S-transferases (GST) compared to *C. elegans*, while tapeworms have a slight gain of mu class GSTs compared to flukes and free-living flatworms (Abad *et al.* 2008; Tsai *et al.* 2013). Overall though, the investigated genomes of parasites do not show any particular expansion of known redox-related genes, except for the antioxidant selenoprotein (Pfam domain SelP_N), which is more commonly occurring in parasites than in free-living species (Supplementary Table S5·2). Given that free-living organisms (exposed to a larger number of more complex metabolic substrates, and other metabolizing organisms) overall are likely to encounter more oxidative stress and xenobiotics than obligate endoparasites do, it would logically follow that free-living organisms should have more complex and varied redox systems. Host-generated oxidative stress may however still be an efficient method to combat parasites, as long as the host has a better developed system of ROS defence (Schirmer *et al.* 1987).

Overall, it appears that antigenic variation of proteins is not a common method in helminths of avoiding the host immune system, and maintaining a chronic infection. Instead, the parasitic worms are using a range of different strategies; minimizing its exposure to the host immune system through encapsulation and other surface modifications and manipulating the host immune system through secretions of immunomodulatory agents. Still, much remains to be understood about these mechanisms, and these helminth genomes provide a platform for accelerating such research.

## UTILIZATION OF HOST RESOURCES

The hosts of parasites often provide a combination of shelter from environmental physical and biological stresses and plentiful and readily accessible food. Since the host has already created a modified metabolome (by food choice, digestion, excretion, etc.), parasites typically encounter a reduced set of potential nutritional substances than free-living organisms do (at least during their parasitic life stages). We can thus predict that parasites adapt by reducing their metabolic capacity to fit the range of available nutrients in the host.

### Modification of the metabolism

The most prominent gene losses in flukes are those of metabolic enzymes, resulting in reduced ability to perform *de novo* synthesis of fatty acids, sterols, cholesterol, purines and amino acids (Berriman *et al.* 2009; Zhou *et al.* 2009; Wang *et al*. 2011). *Clonorchis sinensis* is the only trematode sequenced to date that has all genes encoding enzymes involved in the fatty acid *β*-oxidation pathway (Huang *et al.* 2013). In tapeworms the same losses were predicted, along with the possible loss of the peroxisome organelle from trematodes and cestodes, and further losses of metabolic proteases, amino acid biosynthesis and molybdopterin biosynthesis (Tsai *et al.* 2013; Zheng *et al.* 2013). These gene losses are likely to reflect the morphological changes in these parasites, who have lost their ability to digest in a gut, instead absorbing a simpler spectra of nutrients through its skin (Tsai *et al.* 2013). The loss of digestive metabolism is accompanied by expansions of some tapeworm-specific genes, which appear to aid the absorption and processing of fatty acids, such as fatty acid binding protein (FABP), low-density lipoprotein (LDL) A receptors and the apolipoprotein antigen (Zhou *et al.* 2009; Tsai *et al.* 2013). The main food source for parasitic flatworms is glycogen and in *C. sinensis* a high diversity in key enzymes required for glycolysis, such as hexokinase, enolase, pyruvate kinase, lactate dehydrogenase and phospholipase D has been reported (Wang *et al*. 2011), but our comparative analysis does not find them enriched in comparison to other worm genomes (Supplementary material S5·2, S6). Overall, parasites show few common gained domains – instead domains are more often significantly enriched in free-living worms than in the parasites, such as *α*-N-acetylglucosaminidase (Pfam NAGLU), which breaks down complex sugars (Supplementary material S5·2).

In parasitic nematodes, no correspondingly drastic losses of metabolic enzymes have been reported, even though some studies have performed metabolic reconstruction e.g. (Kikuchi *et al.* 2011; Laing *et al.* 2013; Schwarz *et al.* 2013; Tang *et al.* 2014). An early study did note a lack of enzymes required for *de novo* synthesis of purines, haem and riboflavin in *B. malayi*, but also noted that the complete pathways are present in the symbiotic *Wolbachia*. Many of those enzymes are however also lacking in *Wolbachia*-free *L. loa*, so it is uncertain to what extent the parasites utilizes its *Wolbachia*'s metabolic capacity (Desjardins *et al.* 2013). An explicit comparison of metabolic capacities between *C. elegans, M. incognita, B. malayi* and *Trichinella spiralis* indicated parasites have reduced metabolic capacity, less so in core energy metabolism, but – just as in flatworms (described above) – more pronounced in auxiliary metabolism such as metabolism of co-factors and vitamins (Mitreva *et al.* 2011). Comparisons of EC numbers between free-living nematodes and *H. contortus* discovered some enzyme differences, indicating that their amino acid and carbohydrate metabolism differs (Laing *et al.* 2013). Only nematodes have Pfam domain Ldl_recept_b (LDL B receptors), which are usually present in cholesterol binding proteins, and they seem to be consistently more abundant in animal-parasitic nematodes compared to free-living and plant parasites (Supplementary material S5·2).

In our analysis, we found that there were no GO-terms enriched in secreted proteins in the animal parasites. The few GO-terms enriched in the free-living species, were all metabolic processes, including peptidoglycan catabolic process (GO:0009253), sphingolipid metabolic process (GO:0006665) and cell-wall macromolecule catabolic process (GO:0016998). In nematodes, these enriched GO-terms encompass glycosyl hydrolases, chitinases and N-acetylmuramoyl-L-alanine amidase (a hydrolase breaking down cell-wall glycopeptides) (Supplementary Table S2·1).

For nematodes, rather than losing genes, the main common theme instead seems to be the acquisition of a diverse set of enzymes for digesting complex proteins and carbohydrates, for penetrating and digesting host cells and tissue (Bird *et al.* 2014). Some of these CAZymes (carbohydrate-active enzymes) discovered in *M. incognita* includes GH5 cellulases and xylanases, GH28 polygalacturonases and PL pectate lyases (Abad *et al.* 2008). Both *B. xylophilus* and *Meloidogyne* spp. appear to have acquired a large number of enzymes through horizontal gene transfer (Abad *et al.* 2008; Opperman *et al.* 2008; Kikuchi *et al.* 2011). Although somewhat related in function, the enzymes appear to have been independently acquired from different sources (Kikuchi *et al.* 2011). The ability to acquire metabolic enzymes through horizontal gene transfer does however not seem to be a feature exclusive to plant parasitic nematodes, but is also found as *Wolbachia* insertions in the *D. immitis* genome (Godel *et al.* 2012). HGT in the necromenic *P. pacificus* has been argued to be a pre-adaptation for parasitism by allowing for rapid revolutions in metabolic capacity (Dieterich and Sommer, 2009).

### Communicating using transmembrane proteins

In order to understand how the parasite utilizes the host resources, one of the main groups of proteins to study are the transmembrane proteins. Simple transmembrane proteins can be involved in host–parasite communication, for instance as receptors to host stimuli. More complex transmembrane proteins can allow for selective import/export of substrates between host and parasite. In order to identify any types of transmembrane proteins disproportionally represented in parasites, we performed a GO-term enrichment analysis to look for functions over-represented in proteins with one transmembrane domain, compared to all proteins in each species. By comparing the enriched GO-terms across species we investigated whether any function is more likely to be associated with transmembrane proteins one in parasites than in free-living species. We find that no GO-terms are enriched overall for all parasites, but some GO-terms only occur in parasites, for instance transmembrane receptor protein tyrosine kinase signalling pathway (GO:0007169). In both nematodes and flatworm parasites this GO-term is associated with tyrosine kinase receptors such as furin (protein-activating protease), ephrin receptors (regulation of tissue differentiation) and growth factor receptors (stimulating growth and cell differentiation) (Supplementary Table S9·1, S9·2). That more of these types of proteins have transmembrane domains in parasites could indicate that the parasites are receiving cues from the host regulating its growth and differentiation. Such systems have been described in some detail for individual parasites previously, i.e. (Zheng *et al.* 2013), but we note that this seems to be a cross-phylum parasite adaptation, which repeatedly has occurred in very disparate parasites.

### Concluding remarks

Parasitism is extremely common; it has been estimated that at least half of all animals have at least one parasitic life stage during their life-cycle, and almost all free-living animals are host to many parasitic animals (Price, 1980). Many parasites are not exclusively parasitic, but have free-living life-stages during which they may also be motile, feed and reproduce. Given the many routes to parasitism, and the diversity of parasitic niches that exist, it should perhaps not be surprising that each parasite has undergone its own special adaptations to make it particularly suitable for the environments they encounter throughout their life cycle.

We find several examples of how parasite adaptation is system-specific; it has previously been observed that immunomodulating ES proteins in helminths display a striking diversity, targeting virtually every type of immune cell (Hewitson *et al.* 2009). Evidence emerging from genomes supports this, showing that ES signals change often, resulting in each species having a unique set of ES products. Protease families evolve even more rapidly, with each protease family appearing to be tailored to the specific niche of that species (see above). Helminths do not have obvious systems for antigenic variation, similar to those of viruses, bacteria and single-cell parasites, but they have evolved a rich set of effector proteins, other immunomodulatory methods, and they are using a multitude of surface-modification methods, including cellular and acellular encystment, which allows them to persist in the host for decades. Genes involved in surface tissue formation seem to be general targets for adaptation to parasitism, and thus provides the most convincing example of convergent recruitment of orthologous proteins to similar functions in disparate taxa (*sensu* Christin *et al.* 2010). Adaptations to host metabolism has in tapeworms and trematodes resulted in a spectacular loss of metabolic pathways, which is not matched by nematodes. They instead acquire additional enzymes through horizontal gene transfer. There is however an overall trend of all helminths for losing auxiliary metabolism such as synthesis of co-factors and vitamins, and the peroxisome organelle. Searching for parasite-specific patterns yielded few significant results, but it should be noted that the statistical test is sensitive to the distribution of the character, such that if there is enrichment in only half of the parasites it will not give a significant result, even if there are several independent instances of enrichment. Improved and more sensitive methods for investigating phylogenetically independent parallel evolution are needed to detect those instances, and a separate analysis of only nematodes may identify patterns specific to that phyla. A much larger set of free-living comparator species is also needed to improve the statistical power, but really only of use in nematodes where parasitism has arisen on multiple occasions, or if the investigation is expanded to include the other, quite few, non-helminth bilaterian parasites. Finally, better functional annotation of parasites might also help reveal more parasite-specific characters.

So in spite of a few general common patterns, this study indicates that on a genomic level each evolution of parasitism in helminths has generated many unique adaptations to that specific niche. This leaves us with the challenging problem of having to investigate instances of similar outcomes, generated by very different genomic adaptations, such as the genes involved in formation of the multiple independent inventions of stylets and plant peptide mimics in plant-parasitic nematodes (Bird *et al.* 2014), or effector proteins in helminths (Hewitson *et al.* 2009). Such convergent – but not orthologous – evolution of functions or systems may provide the ultimate answer of what it takes to be a parasite.

## METHODS

In order to do some basic and standardized comparative genomics, predicted proteins were downloaded from all available helminth genomes. For genomes from WormBase, version 241 was used (Table 1, Supplementary Table S1). Functional annotation of proteins was conducted using Interpro v.5.0.7 and KAAS (KEGG Automatic Annotation Server) (Moriya *et al.* 2007; Hunter *et al.* 2012). Pfam and Phobius results were extracted from InterPro results and used to generate Supplementary Tables S2–3, S5–7 and S9. GO-term enrichment was conducted using topGO v.2.12.0 (Adrian and Rahnenfuhrer, 2010), and displayed in Supplementary Tables S2·2, S2·2, S9·1 and S9·2. In order to accelerate the MEROPS annotation process, the MEROPS database (Rawlings *et al.* 2014) was downloaded and blastp searches conducted using the peptides units only, with an *e*-value cut-off of 0·00001 (Altschul *et al.* 1990), and summarized in Supplementary Table S4. For the antigens, all genes with the HSP70 Pfam domain were extracted from the main dataset, and amended with our annotation in Supplementary Table S7. All microexon genes were extracted from supplementary materials (Berriman *et al.* 2009; Tsai *et al.* 2013) and amended with our annotation in Supplementary Table S8.

### Phylogenetic reconstruction

1:1 orthologous genes were isolated using OMA standalone software v.0.99t (Roth *et al.* 2008), and genes in which 25 or more out of the 31 taxa were represented were extracted, results presented in Supplementary Table S10. The extracted clusters were aligned using MAFFT v6.240 (Katoh and Standley, 2014), and conserved blocks were extracted using Gblocks v.0.91b (Castresana, 2000). The resulting alignments were concatenated, and a phylogeny reconstructed using RaxML web server using the Blosum62 model and gamma (Stamatakis *et al.* 2008). The resulting phylogeny is depicted with proportional branch lengths in Fig. 1, and was used for trait correlation testing.

### Trait correlation testing

We examined the associations between mode of parasitism and other traits (the number of domains, number of proteases and enriched GO-terms) using the subroutine phylogenetic generalized least squares (PGLS) in the R-package Caper v. 0.5.2 (Orme, 2013). This method controls for phylogenetic relatedness while determining whether an independent trait (here mode of parasitism) predicts values of another trait (domain frequency). The results were calculated from the normalized and log-transformed values. The significance was assessed using a *t*-test, and the results are reported for protease families and domains in the Supplementary materials. Because of the subjectivity in determining coding the character ‘mode of parasitism’, several different schemes were used, displayed in Supplementary Table S1. The results are inserted to the right in Supplementary Tables S2·2, S4, S6 and S9. Differences in probability (*P*-value) means between datasets were calculated using a paired *t*-test in R 3.0.0 (R Core Team, 2013).

### Invasion secretome analysis

For *S. mansoni*, Supplementary Table S3 was downloaded from (Protasio *et al.* 2012). Transcripts significantly differentially expressed between cercariae and 3 h schistosomula were extracted, and the gene IDs were used to cross-reference with our annotation. Differentially expressed genes that were annotated as proteases or secreted were extracted, and are presented in Supplementary material S3.

## SUPPLEMENTARY MATERIAL

To view supplementary material for this article, please visit http://dx.doi.org/10.1017/S0031182014001449
